# Lipid-Lowering Effects of Tetradecylthioacetic Acid in Antipsychotic-Exposed, Female Rats: Challenges with Long-Term Treatment

**DOI:** 10.1371/journal.pone.0050853

**Published:** 2012-11-30

**Authors:** Silje Skrede, Johan Fernø, Bodil Bjørndal, Wenche Rødseth Brede, Pavol Bohov, Rolf Kristian Berge, Vidar Martin Steen

**Affiliations:** 1 Dr. Einar Martens’ Research Group for Biological Psychiatry, Department of Clinical Medicine, University of Bergen, Bergen, Hordaland, Norway; 2 Center for Medical Genetics and Molecular Medicine, Haukeland University Hospital, Bergen, Hordaland, Norway; 3 The Lipid Research Group, Section for Medical Biochemistry, Institute of Medicine, University of Bergen, Bergen, Hordaland, Norway; 4 Department of Clinical Pharmacology, St Olav’s Hospital, Trondheim, Sør-Trøndelag, Norway; 5 Department of Heart Disease, University of Bergen, Bergen, Hordaland, Norway; University of Santiago de Compostela School of Medicine - CIMUS, Spain

## Abstract

**Background:**

Psychiatric patients often require chronic treatment with antipsychotic drugs, and while rats are frequently used to study antipsychotic-induced metabolic adverse effects, long-term exposure has only partially mimicked the appetite-stimulating and weight-inducing effects found in the clinical setting. Antipsychotic-induced effects on serum lipids are also inconsistent in rats, but in a recent study we demonstrated that subchronic treatment with the orexigenic antipsychotic olanzapine resulted in weight-independent increase in serum triglycerides and activation of lipogenic gene expression in female rats. In addition, a recent long-term study in male rats showed that chronic treatment with antipsychotic drugs induced dyslipidemic effects, despite the lack of weight gain.

**Aims:**

In the current study, we sought to examine long-term effects of antipsychotic drugs on weight gain, lipid levels and lipid composition after twice-daily administration of antipsychotics to female rats, and to investigate potential beneficial effects of the lipid-lowering agent tetradecylthioacetic acid (TTA), a modified fatty acid.

**Methods:**

Female rats were exposed to orexigenic antipsychotics (olanzapine or clozapine), metabolically neutral antipsychotics (aripiprazole or ziprasidone), or TTA for 8 weeks. Separate groups received a combination of clozapine and TTA or olanzapine and TTA. The effects of TTA and the combination of olanzapine and TTA after 2 weeks were also investigated.

**Results:**

The antipsychotic-induced weight gain and serum triglyceride increase observed in the subchronic setting was not present after 8 weeks of treatment with antipsychotics, while lipid-lowering effect of TTA was much more pronounced in the chronic than in the subchronic setting, with concomitant upregulation of key oxidative enzymes in the liver. Unexpectedly, TTA potentiated weight gain in rats treated with antipsychotics.

**Conclusion:**

TTA is a promising candidate for prophylactic treatment of antipsychotic-induced dyslipidemic effects, but a more valid long-term rat model for antipsychotic-induced metabolic adverse effects is required.

## Introduction

The second-generation antipsychotics clozapine (CLZ) and olanzapine (OLZ), arguably the most efficacious pharmacological agents presently recognized for positive symptoms of schizophrenia [1], induce serious metabolic adverse effects such as weight gain, dyslipidemia and glucose dysregulation in patients [2], [3], [4], [5], [6], [7], [8]. Rodent models are frequently used to study antipsychotic adverse effects, with glucose dysregulation consistently reproduced in female and male rats after treatment with both OLZ and CLZ [9], [10], [11], [12]. Increased food intake (hyperphagia) and weight gain is evident in female rats treated with OLZ [7], [13], [14], [15], [16], but not with CLZ [17], [18], [19], although both drugs have orexigenic (appetite-stimulating) potential in the clinical setting [5]. The number of studies concerning the influence of antipsychotic agents on serum lipids is limited in rodents, and the findings have been inconsistent [15], [20], [21], [22]. Recently, however, we repeatedly demonstrated that two weeks of twice-daily oral administration of a constant dose of OLZ (6 mg/kg) elevated fasting serum triglycerides (TG) in female rats [23]. Interestingly, increased serum TG levels were observed both in *ad libitum*-fed rats that gained weight and in food-restricted rats that did not, indicating direct lipogenic effects of OLZ. Our findings were supported by a recent long-term study in male rats, where 40 days of treatment with constant doses (in the range of 2.5–10 mg/kg) of several antipsychotics, including OLZ, elevated postprandial serum TG relative to control, without concomitant increase in body weight [22]. In addition, clinical findings of BMI-independent serum TG increase have been described both in patients treated with CLZ [24] and OLZ [25].

In the present study, we aimed to investigate the effect of the lipid-lowering fatty acid analogue tetradecylthioacetic acid (TTA) on antipsychotic-induced metabolic adverse effects. TTA, a pan-peroxisome proliferator-activated receptor (PPAR) agonist targeting mitochondria, has been demonstrated to reduce body weight gain, adiposity and serum lipids in male rats fed a high-fat diet [26], [27] and to exert lipid-lowering effects both in type 2 diabetes and psoriatic patients [28], [29]. TTA is well tolerated in patients [28], [29], and may represent a novel candidate agent for pharmacological intervention in antipsychotic-treated patients with dyslipidemia. In the present study, we investigate the effect of TTA on body weight, serum lipids and transcriptional changes in peripheral tissues in female rats, both as monotherapy and in combination with antipsychotic drugs.

**Table 1:** Serum levels of antipsychotic agents.

	Subchronic	Chronic
OLZ	0.27±0.03	1.24±0.54
OLZ-TTA	0.83±0.28	0.42±0.64
CLZ		0.10±0.87
CLZ-TTA		0.25±0.06

Fasting serum levels of antipsychotic agents, given in nM, after 2 or 8 weeks of treatment, respectively.

## Materials and Methods

### Ethics Statement

All experiments were approved by and carried out in accordance with the guidelines of the Norwegian Committee for Experiments on Animals (“Forsøksdyrutvalget”, permit number 20102332), and care was taken to ensure minimal animal suffering at all stages of the experiment.

### Animals and Treatment Schemes

Rats were kept under standard conditions with an artificial 12∶12-hour light/dark cycle (lights on: 08∶00) and constant 48% humidity. Animals were allowed access to tap water and free *(ad libitum)* access to standard laboratory chow during the experimental period. Prior to decapitation, rats were anesthetized using isoflurane. Trunk blood was collected in serum tubes, which were left at 4°C for 25–30 minutes and centrifuged at 4°C for 10 minutes (3,000g) to extract serum. Tissue samples were frozen in liquid nitrogen and stored at −80°C until analysis.

**Figure 1:**
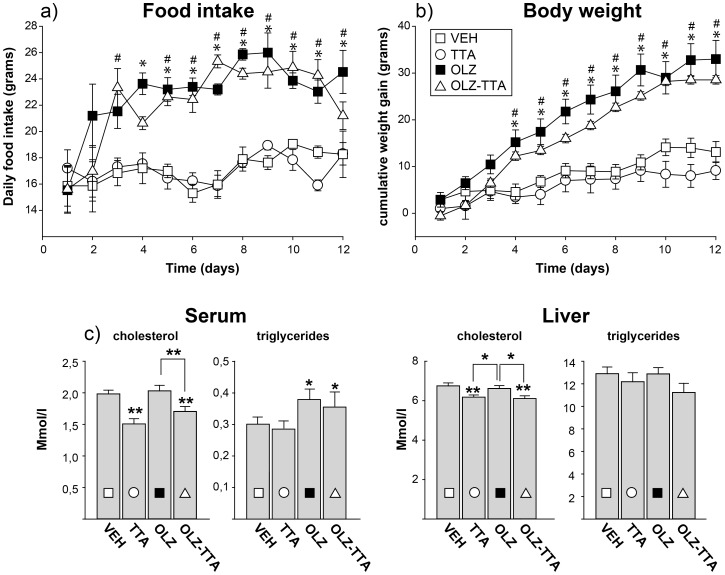
Parameters measured during the subchronic experiment. a) daily food intake, b) daily cumulative weight gain, and c) and serum levels of total cholesterol, triglycerides, liver total cholesterol and triglycerides at the 2-week end point of the subchornic experiment. VEH, vehicle, TTA: tetradecylthioacetic acid, OLZ: olanzapine. * P≤0.05 vs vehicle.

### Subchronic (2-week) Experiment

All antipsychotics were purchased from Toronto Research Chemicals, Inc. (Canada). All stated drug doses equal total amount of drug administered per 24 hours. As previously described [23], female Sprague-Dawley rats (n = 9) housed 3 per cage were administered with vehicle (VEH) or OLZ 6 mg/kg by gavage b.i.d. for 13 days, and sacrificed on day 14 after an overnight fast. As a pilot study on TTA effects, two treatment groups not described in our recent publication [23] were also included in this subchronic experiment: a TTA monotherapy group (150 mg/kg), and a group receiving OLZ (6 mg/kg) and TTA (150 mg/kg) (OLZ-TTA).

**Table 2:** Lipid subspecies in serum in the subchronic experiment.

	VEH	TTA	OLZ	OLZ-TTA
Total FA	100±0	100±0	100±0	100±0
**SFA**	32,7±0,6	32,5±0,5	32,4±0,9	**31,7±0,8** ** ¶
C16∶0	14,5±0,9	15,5±1,1*	15,2±1,2	**15,6±0,4** **
C18∶0	15,9±0,6	15,1±1,0	15,1±1,1	**13,9±0,9** *** ¶
**MUFA**	10,6±1,1	**12,6±1,5** **	12,1±1,6*	**13,1±1,9** **
C16∶1n-7	0,8±0,1	0,7±0,1	1,0±0,3	**1,0±0,3** ¶
C18∶1n-9	6,6±0,8	**9,1±1,3** ***	8,1±1,2*	**9,0±1,5** **
**PUFA n-3**	9,2±0,7	**7,2±0,5** ***	10,3±1,1	**8,5±1,7** ¶
C20∶5n-3 (EPA)	1,9±0,4	**0,9±0,1** ***	2,1±0,6	1,2±0,9
C22∶6n-3 (DHA)	5,7±0,5	**5,0±0,5** **	6,3±0,5*	5,5±0,6
C22∶5n-3 (DPA)	0,9±0,1	**0,7±0,1** **	**1,1±0,2** **	**0,9±0,2** ¶
**PUFA n-6**	47,0±1,1	46,0±1,5	44,9±2,4*	44,2±3,3*
C18∶2n-6 (LA)	18,0±1,6	16,9±1,5	16,9±1,3	**15,8±1,1** **
C20∶4n-6 (AA)	27,7±2,6	27,3±3,0	26,5±2,9	26,8±4,1

Data are given in % of total fatty acid content, and presented as mean%±SEM. * P≤0.05 vs vehicle.

** P≤0.01 vs vehicle.

*** P≤0.001 vs vehicle.

† OLZ-TTA significantly different from TTA, with ¶ P≤0.05, ¶¶ P≤0.01, ¶¶¶ P≤0.001.

**Table 3:** Lipid subspecies in the liver in the subchronic experiment.

	VEH	TTA	OLZ	OLZ+TTA
Total FA	100±0	100±0	100±0	100±0
**SFA**	36,9±0,6	36,6±0,7	36,9±0,6	36,5±0,8
C16∶0	15,1±0,5	14,6±1,1	15,3±0,7	14,6±0,7
C18∶0	20,2±0,8	20,6±1,4	20,1±0,8	20,4±1,0
**MUFA**	9,9±1,2	11,1±1,5	10,8±1,9	11,1±1,8
C16∶1n-7	0,6±0,1	0,5±0,1*	0,8±0,3	**0,7±0,3** ¶
C18∶1n-9	6,7±0,9	8,1±1,4*	7,5±1,4*	8,0±1,4*
**PUFA n-3**	13,3±1,0	**10,9±0,9** ***	14,2±0,8 *	**12,6±1,2** ¶¶
C20∶5n-3 (EPA)	1,2±0,2	**0,5±0,1** ***	1,3±0,4	0,7±0,6
C22∶6n-3 (DHA)	10,0±0,8	9,0±1,0*	10,7±0,6	**10,1±0,5** ¶¶
C22∶5n-3 (DPA)	1,3±0,2	**1,0±0,1** ***	1,4±0,2	**1,2±0,2** ¶¶
**PUFA n-6**	39,6±0,6	40,0±0,6	37,7±2,0*	37,8±2,4* ¶
C18∶2n-6	16,8±1,0	**14,8±1,0** ***	**14,9±1,0** **	**12,9±0,6** ** ¶¶¶
C20∶4n-6	21,1±0,9	**23,4±1,4** **	21,1±2,0	23,1±2,4*

Data are given in % of total fatty acid content, and presented as mean%±SEM. * P≤0.05 vs vehicle.

** P≤0.01 vs vehicle.

*** P≤0.001 vs vehicle.

† OLZ-TTA significantly different from TTA, with ¶ P≤0.05, ¶¶ P≤0.01, ¶¶¶ P≤0.001.

### Chronic (8-week) Experiment

Female Sprague-Dawley rats weighing 240±4 g (mean±SEM) on the first day of treatment were housed individually. Each animal, and chow in each cage, was weighed daily. Self-administration of antipsychotics and TTA was achieved by using peanut butter as a vehicle. Based on previous reports [30], drug doses were lowered compared to the subchronic experiment as the treatment period was extended, in order to determine whether lowered doses over time would result in an extended period of excessive weight gain. The following treatment groups were included: VEH (peanut butter), OLZ 3 mg/kg, aripiprazole (APZ) 3 mg/kg, ziprasidone (ZPS) 3 mg/kg; CLZ 5 mg/kg; TTA 200 mg/kg, OLZ 3 mg/kg+TTA 200 mg/kg, CLZ 5 mg/kg+TTA 200 mg/kg (n = 10 in the VEH-treated group, n = 8 in all other groups). The total daily drug doses were split into two separate administrations (9 am and 2.30 pm). The duration of drug exposure was 55 days, and animals were sacrificed on day 56 (8 weeks) after the initiation of treatment. As in the subchronic experiment, the last drug dose before sacrifice was administered 18–20 hours prior to decapitation. All animals were fasted from 9 pm on the day prior to euthanasia. In order to avoid potential effects of time of day and the duration of fast previous to sacrifice, one animal from each treatment group was sacrificed between 9 am and 11 am on 9 consecutive working days.

**Table 4:** Selected genes with drug exposure-related, differential expression in liver in the subchronic experiment.

	*Scd1*	*Fasn*	*Srebp1*	*Hmgcr*	*Srebp2*	*Ppara*	*Pparg*	*Cpt2*	*Acox1*
VEH [23]	1.00±0.28	1.00±0.12	1.00±0.33	1.00±0.14	1.00±0.09	1.00±0.14	1.00±0.13	1.00±0.11	1.00±0.09
TTA	1.35±0.24	0.97±0.15	**1.47±0.07** **	**0.65±0.09** *	0.80±0.03	1.01±0.08	0.76±0.08	1.27±0.32	**1.77±0.21** **
OLZ [23]	1.25±0.39	0.88±0.14	0.75±0.13	**0.56±0.07** **	0.82±0.10	0.74±0.1	0.72±0.09	0.92±0.30	0.95±0.11
OLZ-TTA	**2.59±0.69** *	0.65±0.11	0.75±0.1	**0.36±0.04** ***	**0.60±0.06** **	0.90±0.04	0.74±0.1	**2.08±0.35** ***	**3.49±0.41** ***

Data are given as fold change relative to vehicle, and presented as mean±SEM. Data shown are normalised to P0 (Arbp), with comparable results when normalised to β-actin.

* P≤0.05 vs vehicle.

** P≤0.01 vs vehicle.

*** P≤0.001 vs vehicle. Subchronic data for the VEH, OLZ, and APZ groups have been reported in a previous article [23]. Gene abbreviations: *Scd1*; stearoyl-CoA desaturase. *Fasn*; fatty acid synthase. *Srebp1*; sterol regulatory element binding protein 1, *Srebp2*; sterol regulatory element binding protein 2. *Hmgcr*; 3-hydroxy-3-methylglutaryl-CoA reductase. *Ppara*: peroxisome proliferator activated receptor α, *Pparg*: peroxisome proliferator activated receptor γ. *Cpt2*: carnitine O-palmitoyltransferase 2. *Acox1*: acyl-Coenzyme A oxidase 1.

### RNA Extraction, cDNA Synthesis and Real-time PCR

Tissue samples (∼30 mg of liver tissue or ∼100 mg of white adipose tissue) were homogenized using a TissueLyser (Qiagen, USA). RNA extraction and cDNA synthesis were performed as previously described [23]. Primers were designed using PrimerExpress (Applied Biosystems, USA) or Primer3 [31]. Relative gene expression levels were determined using the comparative ΔCt method [32].

### Lipid and Glucose Measurements and Fatty Acid Composition Analysis in Serum and in Liver

Levels of TG, phospholipids, and cholesterol in serum and liver were measured enzymatically on the Hitachi 917 system (Roche Diagnostics, Germany), as previously described [23]. Liver lipids were extracted by the method of Bligh & Dyer [23], evaporated under N_2_ and re-dissolved in isopropanol before analysis. Fatty acid composition was analysed by gas-liquid chromatography as previously described [23].

**Figure 2:**
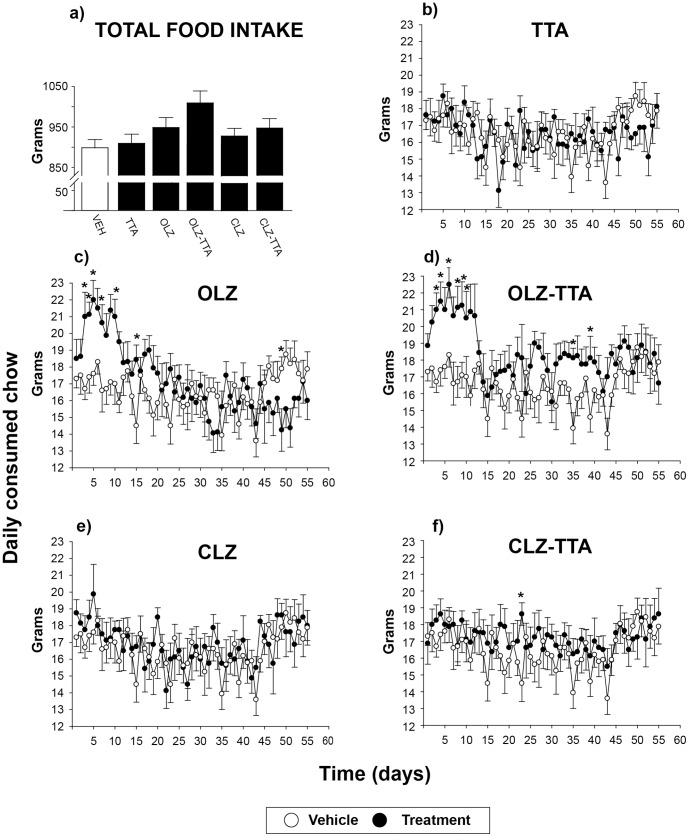
Daily food intake during 8 weeks of treatment with antipsychotics and TTA. Total food intake during the entire treatment period of 8 weeks (a) and daily food intake during chronic treatment with vehicle (open circles), antipsychotic monotherapy, TTA, or antipsychotics in combination with TTA (filled circles; b-f). Data are given as mean±SEM. * P≤0.05 vs vehicle (Dunnett’s post hoc test for daily food intake). TTA: tetradecylthioacetic acid, OLZ: olanzapine, CLZ: clozapine.

**Figure 3:**
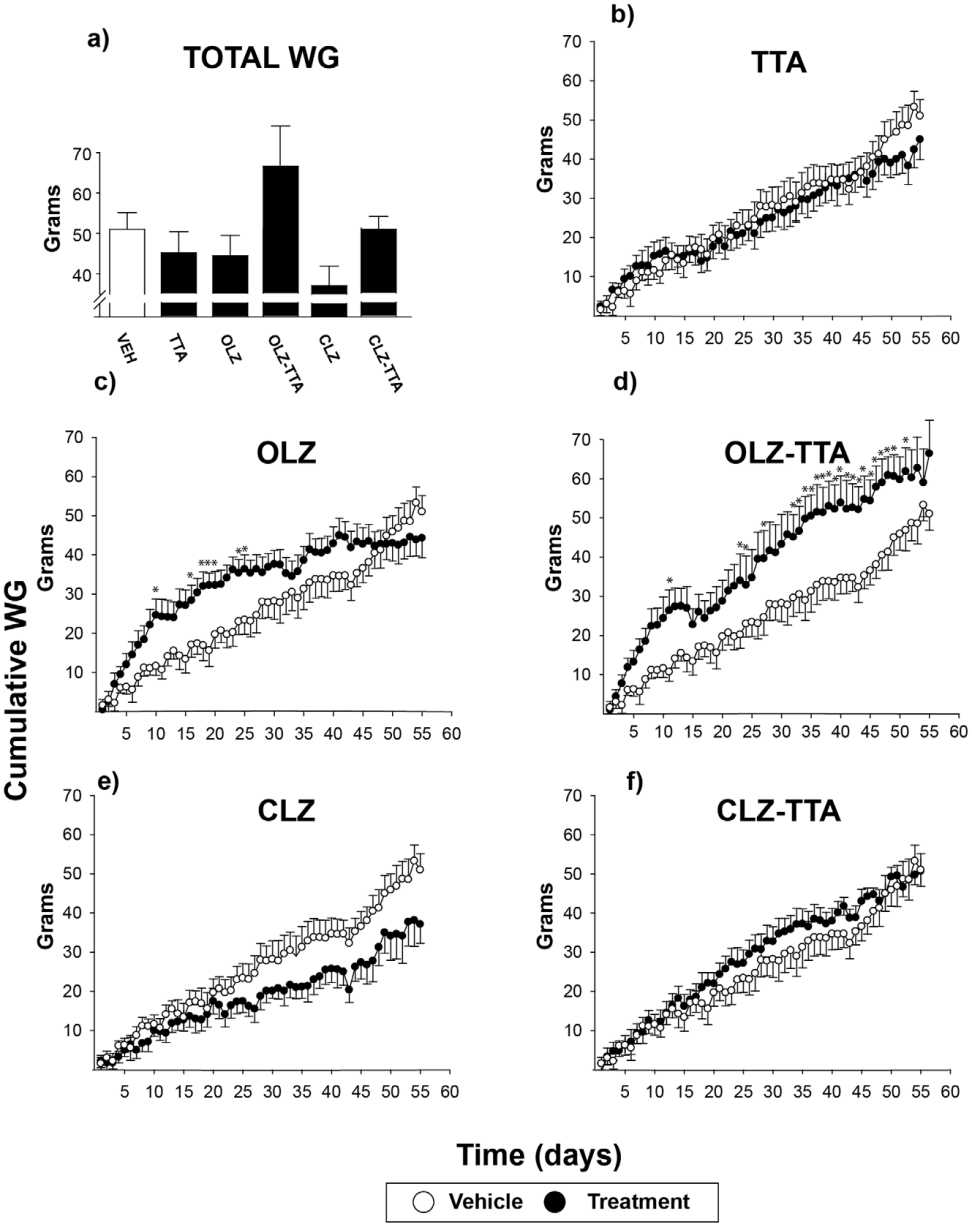
Daily, cumulative weight gain during 8 weeks of treatment with antipsychotics and TTA. Total weight gain during the entire treatment period of 8 weeks (a) and daily cumulative weight gain during chronic treatment with vehicle (open circles), antipsychotic monotherapy, TTA, or antipsychotics in combination with TTA (filled circles; b-f). Data are given as mean±SEM. * P≤0.05 vs vehicle (Dunnett’s post hoc test for daily cumulative weight gain). TTA: tetradecylthioacetic acid, OLZ: olanzapine, CLZ: clozapine.

### Determination of Serum Drug Levels

Serum levels of OLZ, CLZ, APZ, and ZPS (data not shown for the latter two) were determined by means of liquid chromatography/mass spectrometry (LC–MS) at Department of Clinical Pharmacology, St Olavs Hospital, Trondheim, Norway, as previously described [23].

### Hepatic Enzyme Activities

Frozen liver samples were homogenized and post-nuclear fractions were prepared as formerly described [33]. The activity of carnitine palmitoyltransferase (CPT)-II was measured using palmitoyl-CoA and radiolabelled carnitine to yield butanol soluble radiolabelled palmitoylcarnitine [34], [35]. The specific activity of peroxisomal fatty acyl-CoA oxidase (ACOX) was measured as previously described [36], [37].

**Figure 4:**
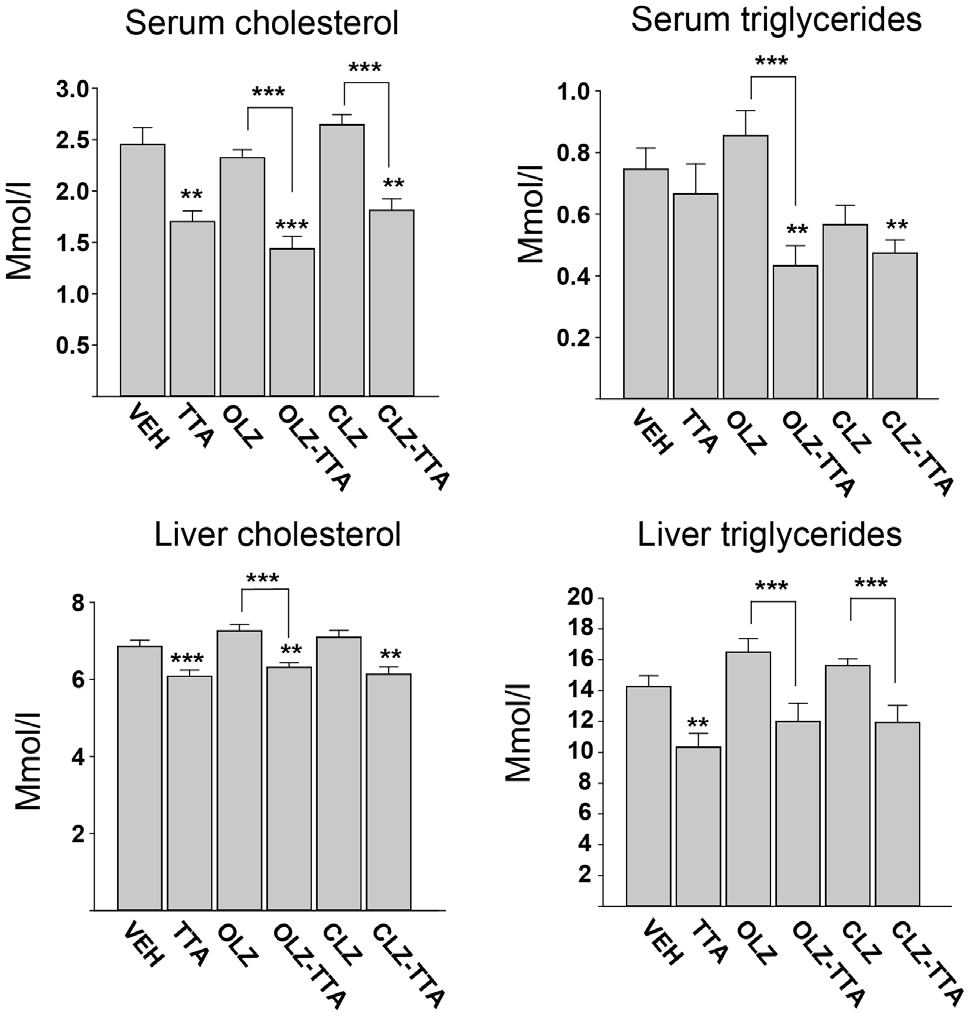
Fasting lipids in serum and liver at 8 weeks. Serum and liver lipids after chronic treatment with vehicle (VEH), olanzapine (OLZ), clozapine (CLZ), tetradecylthioacetic acid (TTA), or a combination of OLZ/CLZ+TTA. * P≤0.05, ** P≤0.01, *** P≤0.001.

**Table 5:** Selected genes with drug exposure-related, differential expression in liver in the chronic experiment.

	*Scd1*	*Fasn*	*Srebp1*	*Hmgcr*	*Srebp2*	*Ppara*	*Pparg*	*Cpt2*	*Acox1*
VEH	1.00±0.36	1.00±0.26	1.00±0.09	1.00±0.14	1.00±0.11	1.00±0.07	1.00±0.10	1.00±0.10	1.00±0.10
TTA	**2.22±0.36** *	0.97±0.20	1.08±0.18	**0.57±0.06** **	0.84±0.12	1.14±0.11	**1.77±0.21** **	**1.38±0.09** **	**2.23±0.30** ***
OLZ	1.15±0.17	0.70±0.16	**0.58±0.06** ***	0.78±0.09	0.86±0.08	1.21±0.13	1.05±0.12	0.92±0.06	0.93±0.08
OLZ-TTA	**2.73±0.76** *	0.81±0.12	**1.57±0.15** **	**0.55±0.09** *	**0.44±0.05** ***	0.91±0.12	**2.51±0.37** ***	**1.55±0.11** ***	**3.39±0.47** ***
CLZ	1.57±0.98	0.60±0.10	0.77±0.15	0.90±0.27	0.73±0.11	**1.41±0.13** **	1.20±0.23	0.93±0.07	0.82±0.12
CLZ-TTA	**2.74±0.47** **	0.57±0.10	1.12±0.14	**0.50±0.08** **	**0.51±0.07** ***	**1.39±0.17** *	**2.10±0.19** ***	**1.83±0.11** ***	**2.47±0.34** ***
APZ	0.60±0.20	0.44±0.07	**0.70±0.10** *	**0.43±0.04** ***	0.77±0.07	**1.33±0.16** *	1.07±0.22	**0.73±0.05** *	**0.64±0.06** *
ZPS	1.55±0.74	0.57±0.10	**0.50±0.07** ***	0.99±0.14	0.92±0.12	1.29±0.18	1.07±0.13	1.01±0.12	0.90±0.18

Data are given as fold change relative to vehicle, and presented as mean±SEM. Data shown are normalised to P0 (Arbp), with comparable results when normalised to β-actin.

* P≤0.05 vs vehicle.

** P≤0.01 vs vehicle.

*** P≤0.001 vs vehicle. For abbreviations, see legend to Table 3.

### Statistical Analysis

Food intake was analyzed using two-way repeated measures ANOVA, with treatment as between-subject variable and time as within-subject variable in both experiments. In the chronic experiment, weeks were used as time variable instead of days, avoiding excessive degrees of freedom. Body weight changes were analyzed using the same method. In the 2-week (subchronic) experiment, the analysis included 4 treatment groups (VEH, OLZ, TTA, and OLZ-TTA), whereas the chronic experiment contained 8 treatment groups (VEH, APZ, ZPS, TTA, CLZ, CLZ-TTA, OLZ, OLZ-TTA). Tukey’s post hoc test was applied in order to determine all significant inter-group differences, while Dunnett’s post hoc test was utilized when comparing all treatment groups with VEH. To detect differences at specific time points throughout the time-course, one-way ANOVA analysis with subsequent post-hoc tests was performed. For all other comparisons, we used two-sided Student’s t-test. P values ≤0.05 were considered statistically significant. All tests were conducted with PASW Statistics Version 18 (PASW statistics; SPSS, USA).

**Table 6:** Relevant genes examined in brown adipose tissue (BAT) in the subchronic study.

	*Ppargc1a*	*Ppargc1b*	*Ucp1*	*Ucp3*	*Ppara*
VEH [23]	1±0.20	1±0.09	1±0.16	1±0.12	1±0.06
TTA	0.85±0.23	1.20±0.07	0.79±0.11	1.19±0.14	1.18±0.04
OLZ [23]	**0.41±0.10** *	1.18±0.09	**0.49±0.04** **	0.90±0.05	1.33±0.15
OLZ-TTA	**0.30±0.05** **	1.08±0.08	**0.57±0.08** *	1.18±0.20	0.96±0.06

Data are given as fold change relative to vehicle, and presented as mean±SEM. Data shown are normalised to P0 (*Arbp*), with comparable results when normalised to *β-actin*.

* P≤0.05 vs vehicle.

** P≤0.01 vs vehicle.

*** P≤0.001 vs vehicle.

**Table 7:** Relevant genes examined in brown adipose tissue (BAT) in the chronic study.

	*Pgc1a*	*Pgc1b*	*Ucp1*	*Ucp3*	*Ppara*
VEH	1±0.14	1±0.11	1±0.18	1±0.13	1±0.11
TTA	**0.63±0.08** *	0.90±0.09	**0.48±0.07** *	0.87±0.16	0.90±0.09
OLZ	0.76±0.24	0.85±0.06	0.53±0.08	0.87±0.07	0.85±0.06
OLZ-TTA	**0.34±0.04** ***	0.83±0.08	0.98±0.19	0.98±0.09	1.00±0.08
CLZ	0.46±0.09	1.01±0.08	1.26±0.17	1.33±0.10	0.83±0.08
CLZ-TTA	0.66±0.26	0.85±0.05	0.85±0.14	0.91±0.09	0.85±0.05
APZ	0.62±0.13	0.96±0.03	1.04±0.21	0.95±0.06	0.96±0.03

Data are given as fold change relative to vehicle, and presented as mean±SEM. Data shown are normalised to P0 (*Arbp*), with comparable results when normalised to *β-actin*.

* P≤0.05 vs vehicle.

** P≤0.01 vs vehicle.

*** P≤0.001 vs vehicle.

## Results

### Serum Drug Levels

In both the subchronic and the chronic experiments, serum levels of antipsychotic agents at the time of dissection, i.e. 18–20 hours after administration of the last drug dose, were in the lower nM range (Table 1). No consistent effect of TTA on OLZ or CLZ levels was observed across the two experiments (Table 1).

### Impact of Subchronic Exposure to Antipsychotics and TTA on Food Intake, Body Weight Gain and Serum Lipids in Female Rats

In a subchronic experiment we investigated how 2 weeks of treatment with the lipid-lowering agent TTA affected the previously demonstrated OLZ-induced weight gain and serum triglyceride increase in female rats [23], with published data from the OLZ treatment included here for comparison. Four treatment groups were included in the analyses; 1) Vehicle (VEH), 2) TTA, 3) OLZ, and 4) a combination of OLZ and TTA (OLZ-TTA). Repeated-measures two-way ANOVA analysis was performed on average daily food intake (3 rats per cage) and individual daily cumulative weight gain, with treatment and time as factors. A significant main effect of treatment was evident for both food intake [F(3, 8) = 16.30, p<0.01] (Figure 1A) and weight gain [F(3, 32) = 11.24, p<0.001] (Figure 1B), with a Tukey’s post hoc test revealing a significant elevation (p>0.05) by OLZ, but not by TTA, relative to vehicle. Concordantly, TTA reduced neither food intake nor weight gain in the OLZ-TTA treatment group relative to OLZ.

Even though effects of TTA on food intake and body weight were negligible, we investigated whether lipid-lowering effects of TTA were present. Indeed, subchronic TTA treatment significantly (p<0.05) reduced serum cholesterol and phospholipid levels relative to VEH, evident both in the TTA and in the OLZ-TTA treatment groups. In contrast, TTA did not reduce serum triglyceride (TG) or free fatty acid (FFA) levels. Furthermore, serum lipid levels in the TTA-OLZ treatment group were not significantly lower than in the OLZ treatment group, in which TG levels were elevated relative to control. In the liver, TTA induced a significant decrease in cholesterol levels, as well as a non-significant trend towards decreased TG levels, evident both in the TTA and in the OLZ-TTA treatment groups (Figure 1D).

### Fatty Acid Composition in Serum and Liver after Subchronic Treatment with OLZ and TTA

Despite moderate effects of TTA on total TG and FFA serum levels, the lipid-lowering effects observed for cholesterol and phospholipids suggested that the dose used for TTA (150 mg/kg) was sufficient to induce effects in the female rat. To gain further insight into how TTA affects fatty acid metabolism, we examined the effect on fatty acid (FA) composition, i.e. levels and subspecies of saturated FAs (SFAs), monounsaturated FAs (MUFAs) and polyunsaturated FAs (PUFAs). Both OLZ and TTA affected the amounts of major lipid species, both in serum and in the liver (Tables 2 and 3). Of note, the lipid-lowering TTA and the lipogenic OLZ induced similar effects on several lipid species, particularly evident for MUFAs and specifically with respect to the elevation of oleic acid (C18∶1n-9) (Tables 2 and 3). TTA and OLZ also had opposite effects on some lipid entities, such as the n-3 PUFAs EPA, DHA and DPA, which were reduced by TTA and elevated or displayed a trend towards elevation by OLZ (Tables 2 and 3).

### Drug-induced Transcriptional Alterations in Lipid Metabolism Genes

The sterol regulatory element binding protein (SREBP) is a master regulator of cholesterol and fatty acid biosynthesis [38], previously demonstrated to be activated by antipsychotic drugs [39], [40], [41]. OLZ-induced, SREBP-controlled lipogenic effects have been observed both in cultured cells [39] and *in vivo*, i.e. in white adipose tissue [23], [42] and liver [12] in the rat. In order to identify mechanisms by which OLZ and TTA affect serum and liver lipid levels, we investigated transcriptional effects on genes involved in lipid metabolism in the liver, but in the subchronic experiment, transcriptional effects of OLZ in the liver were minor (Table 4). The effects of TTA on lipogenic gene expression were also moderate. However, a marked upregulation (mean%±SEM; 259±69%, P<0.05) of *Scd1*, encoding the stearoyl-delta 9 desaturase (SCD1) that converts stearic acid (C18∶0) to oleic acid (C18∶1n-9), was observed in the OLZ-TTA treatment group (Table 4). In agreement with the cholesterol-lowering effect of TTA, hepatic levels of *Hmgcr,* the rate-limiting step in cholesterol biosynthesis, was downregulated both in the TTA (65±9%, P≤0.05) and OLZ-TTA (36±4%, P≤0.001) treatment groups, but also in the OLZ treatment group (56±7%, P≤0.05), despite no clear effect of OLZ on serum cholesterol levels. *Srebf2*, encoding the SREBP2 transcription factor – the main regulator of cholesterol biosynthesis genes – was significantly downregulated in the OLZ-TTA treatment group (60±6%, P≤0.01), with a trend towards downregulation in the TTA group (80±3%, P = 0.06). TTA has been suggested to mediate its lipid-lowering effect via increased hepatic β-oxidation through PPARα-controlled transcriptional activation [26], [27]. We therefore investigated hepatic expression levels of the PPARα-controlled genes *Acox1,* encoding the rate-limiting step of peroxisomal fatty acid β-oxidation, and *Cpt2*, involved in mitochondrial fatty acid oxidation. Hepatic *Acox1* expression was significantly upregulated both in the TTA treatment group (177±21%, P≤0.01) and, with a more pronounced effect, in the OLZ-TTA treatment group (349±41%, P≤0.001). A similar pattern was observed for *Cpt2* (Table 4), albeit reaching statistically significance (P<0.001) only in the OLZ-TTA treatment group. OLZ monotherapy did not induce significant effects on *Acox1* or *Cpt2*.

Since lipase expression levels may contribute to alterations in lipid levels, we measured drug effects on lipase genes, such as adipose triglyceride synthase (*Pnpla2*/*Atgl*) and hormone-sensitive lipase (*Hsl*), in visceral (parametrial) white adipose tissue (WAT). However, with the exception of a slight OLZ-induced downregulation of *Pnpla2* in parametrial WAT, no significant alterations were observed.

### Long-term Effects of Antipsychotics and TTA on Food Intake, Body Weight and Lipid Parameters

TTA-induced effects are typically more evident after long-term than after subchronic treatment [26]. The moderate effects observed in our subchronic experiment may thus be due to lack of sufficient exposure time and/or dosage. The effect of a moderately higher TTA dose on metabolic parameters in female rats was therefore investigated in a chronic setting. Weight-inducing effects of long-term antipsychotic treatment normally follows an opposite pattern in rat, with declining rates of weight gain in the long-term setting [30], [43]. However, in light of the weight-independent elevation of serum triglycerides recently shown after chronic treatment in male rats, female rats were treated chronically (8 weeks) with either atypical antipsychotic drugs (OLZ, CLZ, APZ, or ZPS), TTA, or TTA in combination with the orexigenic atypical antipsychotics OLZ or CLZ (OLZ-TTA, CLZ-TTA). As expected, the effect of antipsychotic drugs on food intake and weight gain was most prominent during the first weeks of treatment, followed by a decline, with resultant lack of significant differences in total cumulative weight gain at the experiment’s 8-week end point. However, a two-way repeated-measures ANOVA analysis for the entire treatment period (8 weeks), with treatment and time (with weeks replacing days in order to avoid excessive degrees of freedom) as factors, revealed a statistically significant main effect of treatment on cumulative weekly food intake [F(4,58)  = 3.66, P≤0.01] and cumulative weekly weigh gain [F(7,58)  = 3.78, P<0.01]. Dunnett’s post-hoc tests revealed significant effects with respect to food intake for OLZ (P≤0.05) (Figure 2C) and OLZ-TTA (P≤0.01) (Figure 2D), as well as a trend towards statistically significant effect for APZ (P = 0.10) (data not shown), relative to VEH-treated controls. With respect to weight gain, Dunnets’ post-hoc test revealed only the OLZ-TTA treatment group as significantly (P≤0.01) different from VEH. Accordingly, analysing each time point separately by a one-way ANOVA, the only significant increase in weight gain at the late time points occured in the OLZ-TTA treatment group relative to VEH (Figure 3D). It should also be noted that there seems to be a clear trend towards potentiation of body weight gain in the co-treatment groups OLZ-TTA and CLZ-TTA relative to the OLZ and CLZ monotherapy groups (Figure 3C-F), although Tukeys’ post-hoc test revealed no significant effects.

In the chronic setting, the lipid-lowering effects of TTA were much more evident than in the subchronic setting, with marked reduction of trends towards reduction for all major lipid classes, both in liver and in serum (Figure 4). Quite the opposite, none of the antipsychotic drugs significantly altered the levels of any serum or liver lipid classes (Figure 4). Despite the lack of antipsychotic-induced effects, the effects of TTA on serum TG in the co-treatment groups (OLZ-TTA and CLZ-TTA) were more pronounced than in the monotherapy groups, reminiscent of TTA’ potentiating effect on weight gain in OLZ-treated rats.

### Transcriptional Changes in the Liver

Like in the subchronic setting, only minor hepatic lipogenic transcriptional alterations were observed for the antipsychotic drugs, with a trend towards downregulation for both SREBP1 and SREBP2 targets genes (Table 5). TTA also had negligible effects on SREBP1 target genes in the liver, with the exception of *Scd1*, which was strikingly upregulated in all TTA treatment groups (Table 5). Reminiscent of the subchronic setting, TTA induced downregulation of SREBP2-controlled cholesterol biosynthesis genes. TTA’s stimulatory effect on the transcription of PPARα and its target genes in the liver was also maintained in the chronic setting, with significant upregulation of *Acox1* after treatment with TTA (222±30%, P≤0.001), OLZ-TTA (339±47%, P≤0.001) and, furthermore, in the CLZ-TTA group (247±34%, P≤0.001) (Table 5). Corresponding transcriptional effects were observed for *Cpt2* expression (Table 5). Enzymatic activity corroborated the transcriptional changes (Figure S1).

### Transcriptional Alterations in White and Brown Adipose Tissues

In contrast to the pronounced OLZ-induced transcriptional activation of lipogenic genes observed in visceral WAT in the subchronic setting, hypothesized by us to be of relevance to the observed increase in serum TG levels [23], chronic exposure induced only minor transcriptional effects in parametrial WAT. Of note, *Scd1* was significantly downregulated by OLZ (29±12%, P≤0.05) and a trend towards downregulation was observed in the OLZ-TTA treatment group (40±9%, P = 0.09), demonstrating opposite effects to what we observed in the subchronic setting. No significant effects were observed for TTA monotherapy. The expression of key lipase genes ((*Pnpla2*/*Atgl* and *Hsl*) was unaffected in WAT in the chronic experiment (data not shown).

In order to investigate whether reduced energy expenditure could contribute to the observed differences, or trends towards differences, in weight gain between some of the treatment groups, we examined brown adipose tissue (BAT) expression levels of several thermogenic markers, among them peroxisome proliferative activated receptor gamma coactivator 1 alpha (PGC1α, encoded by *Ppargc1a*) and uncoupling protein 1 (*Ucp1*). In the subchronic experiment, TTA monotherapy did not have significant effects on the expression of key thermogenic markers in BAT (Table 6), while OLZ treatment downregulated *Ppargc1a* and *Ucp1*, an effect that was reflected in the OLZ-TTA group. After chronic treatment, *Ppargc1a* downregulation was observed both in animals receiving TTA and in the OLZ-TTA group, but not in the OLZ group (Table 7).

## Discussion

In this study, we evaluated the modified fatty acid TTA as a potential pharmacologic intervention strategy for metabolic adverse effects of antipsychotic agents, primarily the metabolically potent drugs OLZ and CLZ. TTA was initially examined in a subchronic (two-week) experiment, as OLZ had previously been shown to induce weight gain and elevated fasting triglyceride levels in female rats within this time frame [23]. Two weeks of TTA exposure reduced serum cholesterol and phospholipid levels in female rats, but had no effect on serum TG or FFA levels. More importantly, TTA failed to prevent weight gain and elevated serum TG levels induced by subchronic OLZ exposure. However, it should be noted that metabolically relevant effects of pharmacological agents do not necessarily depend on absolute amounts of lipids, but also on lipid composition [44]. Interestingly, despite the fact that TTA did not reduce absolute levels of serum TG and FFA in the subchronic setting, the amounts of several fatty acid subspecies were affected by TTA. Such effects were also observed for OLZ, with differential effects of TTA and OLZ on some lipid subspecies, e.g. n-3 PUFAs, but with comparable effects on other FAs, such as MUFAs. For instance, both TTA and OLZ increased serum levels of the delta-9-desaturase product oleic acid (C18∶1n-9) and decreased its precursor stearic acid (C18∶0), in line with previous studies demonstrating increased 18∶1/18∶0 ratio both for OLZ [22], the second-generation antipsychotic risperidone [45] and TTA [46]. The 18∶1/18∶0 ratio is an index of increased activity of stearoyl CoA (delta-9) desaturase (SCD1) [47], [48], encoded by the SREBP1 target gene *Scd1*.

Despite the elevated 18∶1/18∶0 ratio, *Scd1* expression was not significantly increased by TTA or OLZ monoterapy, while significantly elevated in the OLZ-TTA treatment group. This effect was not observed for any of the other SREBP1-controlled fatty acid biosynthesis genes, suggesting that the effect on *Scd1* expression was mediated by mechanisms additional to SREBP activation. Indeed, *Scd1* has also been classified as a PPARα-target gene [49], and it is possible that the *Scd1* upregulation observed in in our experient results from a combination of SREBP1 and PPARα activation. It should be noted that the presence of elevated 18∶1/18∶0 ratio with concomitant lack of elevated *Scd1* expression in the liver was also observed after chronic treatment with OLZ [22].

The lack of TTA effects on total TG and FFA serum levels following subchronic exposure in female rats contrasts previous studies in male rats [26], likely due to the relatively short duration of treatment. We therefore investigated the effect of TTA in female rats in a chronic (8-week) setting. Indeed, the increased duration of treatment had significant effects on several parameters, with reduced lipid levels, including TG and FFA, in all treatment groups that included TTA (TTA, OLZ-TTA and CLZ-TTA), apparently with more pronounced lipid-lowering effects of TTA in the co-treatment groups. Such a co-treatment effect was also evident for weight gain, with a trend towards elevated weight gain in OLZ-TTA and CLZ-TTA treatment groups relative to OLZ and CLZ groups, respectively. At first glance, the co-occurrence of the potentiated weight gain and serum lipid reduction in the antipsychotic-TTA co-treatment groups may seem paradoxical. However, according to the adipose tissue expandability hypothesis, metabolically unfavourable effects on serum lipid levels is not an inevitable consequence of obesity [50]. In fact, up to a certain threshold, expanding WAT tissue mass may provide storage capacity for lipids, thus reducing serum lipid levels [50]. In line with this, a genetically induced increase in WAT storage capacity in mice has been shown to lead to a healthy serum lipid profile, despite massive weight gain [51]. Whether this phenomenon occurred in our experiment remains uncertain, since only negligible alterations in the expression of lipid biosynthesis and storage genes, none of which indicated increased lipid storage capacity, were observed in WAT.

Although time-dependent decline of OLZ-induced hyperphagia and weight gain in the rat has previously been described [16], [30], [43], the lack of dyslipidemic effects in chronically exposed rats contrasted our subchronic findings as well as a recent chronic study in male rats, where postprandial serum TG levels were elevated after 40 days of treatment with OLZ as well as other antipsychotics [22]. Gender-specific effects may partially account for the observed differences, possibly with gradual tolerance towards antipsychotics in female rats. If so, this effect seems to be specific to antipsychotic drugs, since the effects of TTA were more pronounced after eight than after two weeks of treatment in female rats. Differing nutritional status (fasted vs. postprandial) is another important distinction between the two chronic studies. Finally, it should be noted that during the last two weeks of our experiment, weight gain surged in VEH-treated female rats, possibly occluding other antipsychotic-induced metabolic adverse effects. The pronounced lipid-lowering effect of TTA found in the OLZ-TTA and the CLZ-TTA treatment groups is promising with respect to the potential role of TTA in preventing antipsychotic-induced hyperlipidemia. As the antipsychotic drugs induced neither weight gain nor dyslipidemic effects in the chronic setting, however, no firm conclusion can be drawn regarding TTA’s potential clinical value at this point. An optimized model for antipsychotic-induced dyslipidemia is warranted to conclude about the lipogenic effects of atipsychotics, as well as potential clinical benefits of TTA.

The trends towards more pronounced effects on metabolic parameters in the antipsychotic-TTA treatment groups, as observed for serum lipids, was also evident with respect to body weight gain and to alterations in gene expression. The mechanisms underlying these add-on effects are unclear. Reduced thermogenic activity in BAT, previously demonstrated in OLZ-treated rats [52], may be of relevance for the observed metabolic changes. Indeed, the expression levels of the thermogenic marker *Ucp1* was reduced in both the OLZ and TTA monotherapy groups, but not in the co-tratment group, failing to provide an explanation for the potentiated weight gain in this group. Although TTA had no consistent effect on OLZ and CLZ serum levels, it is tempting to speculate that TTA may increase the bioavailability of antipsychotics in the rat through altering the drugs’ pharmacokinetics or other aspects of antipsychotic turnover in the body. In humans, OLZ and CLZ are primarily metabolized by CYP1A2 [53], which does not play an important role in the metabolism of TTA [54]. Thus, the theoretical basis of inhibition or induction of antipsychotic metabolism by TTA is not present, but no experimental evidence presently exists in this regard. TTA may still affect pharmacodynamic aspects of antipsychotic drug metabolism, such as tissue distribution.

In conclusion, based on the marked lipid-lowering effects of TTA in female rats, particularly evident when co-treated with the orexigenic antipsychotic drugs OLZ and CLZ in a chronic setting, the potential of TTA as a candidate for pharmacological intervention of antipsychotic-induced dyslipidemic effects is present. However, in order to make firm conclusions, a refined rodent model for antipsychotic-induced dyslipidemia must be available, along with an improved understanding of how factors such as gender, nutritional status, mode of drug administration and drug doses affect the phenotype. Our findings lend further support to the notion that antipsychotic-induced weight gain and dyslipidemia may occur independently, which should be taken into account when treatment regimes for metabolic disturbances is developed.

## Supporting Information

Figure S1 **Expression levels and activity of key oxidative enzymes in liver at 8 weeks.** Expression and activity of acyl-Coenzyme A oxidase 1 (Acox) and carnitine O-palmitoyltransferase 2 (CPT2) in liver from chronically treated rats. * P≤0.05, ** P≤0.01, *** P≤0.001. ¶ P≤0.05 vs TTA, ¶¶ P≤0.01 vs TTA.
(TIF)
