# Supplementary figures and images for: Lipid-Lowering Effects of Tetradecylthioacetic Acid in Antipsychotic-Exposed, Female Rats: Challenges with Long-Term Treatment
Source: PLoS One. 2012 Nov 30;7(11):e50853. doi: 10.1371/journal.pone.0050853 (PMC3511315; doi:10.1371/journal.pone.0050853)

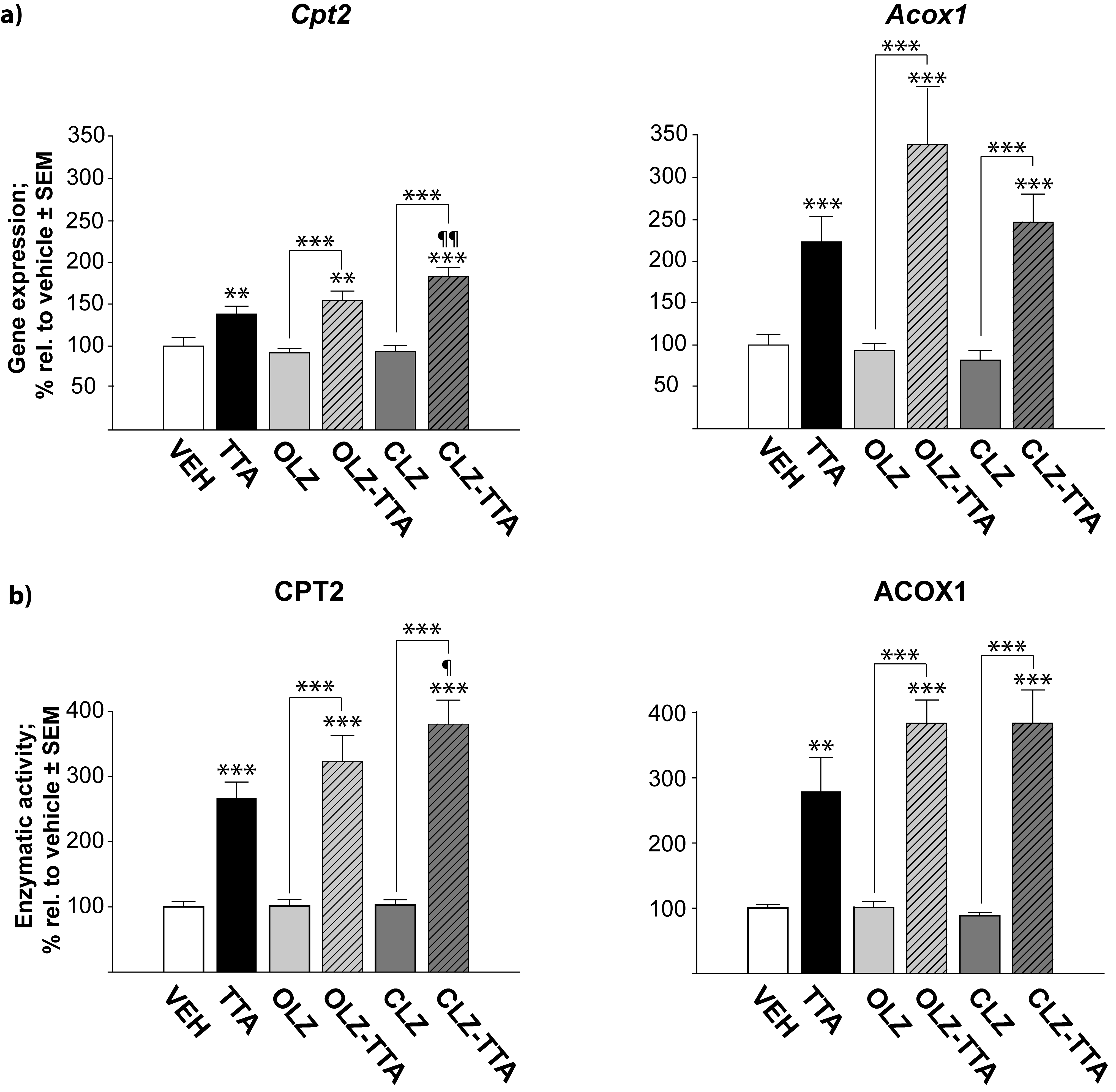

Supplement: Figure S1 — Expression levels and activity of key oxidative enzymes in liver at 8 weeks. Expression and activity of acyl-Coenzyme A oxidase 1 (Acox) and carnitine O-palmitoyltransferase 2 (CPT2) in liver from chronically treated rats. * P≤0.05, ** P≤0.01, *** P≤0.001. ¶ P≤0.05 vs TTA, ¶¶ P≤0.01 vs TTA. (TIF) [file pone.0050853.s001.tif]
